# The accuracy of prenatal diagnosis of selective fetal growth restriction with second trimester Doppler ultrasound in monochorionic diamniotic twin pregnancies

**DOI:** 10.1371/journal.pone.0255897

**Published:** 2021-08-09

**Authors:** Yao Wang, Ai Zhang, Tineck Stock, Enrico Lopriore, Dick Oepkes, Qiuzhen Wang

**Affiliations:** 1 Public Health School, Medical College of Qingdao University, Qingdao, China; 2 Qingdao Women and Children’s Hospital, Qingdao University, Qingdao, China; 3 Department of Obstetrics, Leiden University Medical Center, Leiden, The Netherlands; 4 Division of Neonatology, Department of Pediatrics, Leiden University Medical Center, Leiden, The Netherlands; Medicina Fetal Mexico, MEXICO

## Abstract

**Background:**

Selective fetal restriction growth (sFGR) is one of the common diseases of monochorionic diamniotic (MCDA) twin pregnancies, resulting in many adverse outcomes. At present, second trimester ultrasonography is widely used in the prenatal diagnosis of sFGR, but the diagnostic effectiveness is still uncertain. The aim of this study is to assess the diagnostic accuracy of second trimester Doppler ultrasound measurements for sFGR.

**Methods:**

A retrospective study included 280 pregnant women (118 with and 162 without sFGR) with MCDA pregnancies was conducted in the fetal medicine center from Leiden University Medical Center from January 2008 to December 2013. The women participating had already undergone an ultrasound examination in the second trimester. The postnatal criteria of sFGR was a single birth weight (BW) < 3 rd percentile in a twin, or birth weight discordance (BWD)≥25% between two twins, while the BW of the smaller twin < 10th percentile. Early prenatal diagnosis of sFGR was defined as a single EFW < 3 rd percentile in a twin, or at least 2 of the following 4 parameters must be met (fetal weight of one fetus < 10th percentile, AC of one fetus <10th percentile, EFW discordance≥25%, UA pulsatility index (PI) of the smaller fetus > 95th percentile). According to the diagnosis of sFGR after birth, we evaluate diagnostic effectiveness of Doppler ultrasound in the second trimester for sFGR.

**Results:**

Of these 280 participants, the mean age was 32.06 ± 4.76 years. About 43.9% of pregnant women were primiparas. The ability of second trimester Doppler ultrasound to accurately diagnosed sFGR is 75.4%, missed diagnosis rate and the misdiagnosis rate were 24.6% and 10.5% respectively. The ROC curve indicated that the combination of AC discordance, EFW discordance, and small fetal UA blood flow was the best diagnostic indicator of sFGR in MCDA pregnancy with the AUC was 0.882 (95%CI, 0.839–0.926).

**Conclusions:**

Second trimester Doppler and ultrasound measurements is an effective method for early prenatal diagnosis of sFGR. The combined indicator of AC discordance, EFW discordance, and the small fetal UA blood flow reaches highest diagnostic value.

## Introduction

Selective fetal growth restriction (sFGR) is recognized as a severe complication of monochorionic diamniotic (MCDA) twin pregnancy, resulting in intrauterine fetal death, and long-term neurological or cognitive impairment [1, 2]. As the increasing incidence of twin pregnancy, it is of great importance to prenatal diagnose the high risk twins in order to reduce the adverse perinatal outcomes of MCDA pregnancy.

Fetal growth is the result of a combination of factors, of which the placenta is essential for the sufficient transfer of gases, nutrients and metabolites between mother and fetus [3]. The studies has indicated that sFGR in MCDA twins is closely related to unequal distribution of the placenta and distribution of blood through placental anastomosis, which means that fetal blood supply may be related to increased risk of adverse birth outcomes [4, 5].

The umbilical artery (UA) is an important vascular pathways, playing a pivotal role in fetal growth. UA Doppler blood flow is a parameter that best reflects the differences in intrauterine growth restriction of MCDA twins [6]. But, as reported in a previous study, abnormal end diastolic blood flow of UA was usually observed in small twins with marked inter-twin weight discordance [7]. This means that the ability to detect the risk of sFGR by monitoring UA in twins with less weight discordance is limited. In addition, since only severe fetal hypoxemia could cause the typical abnormal Doppler velocity waveform, it may not be possible to determine the mild perfusion impairment before the onset of fetal damage in time [8].

Currently, Doppler and ultrasound is conventionally used in the prenatal diagnosis of sFGR twins through fetal abdominal circumference (AC), estimated fetal weight (EFW) in contemporary obstetric clinic practice [9]. However, compared with singleton pregnancy, the accuracy of using ultrasound to assess twins’ EFW is lower [10]. On the other hand, twins are known to have lower birth weights than the average level, these are not pathological fetuses but it is very difficult to distinguish them [11].

The importance of ultrasound assessment during the second trimester for sFGR has been emphasize by international guidelines. The guidelines recommend that MCDA twin pregnancy should undergo the routine Doppler ultrasound scan every two weeks from 16 weeks’ gestation, recording the fetal biological characteristics, and calculating the EFW discordance [9, 12]. However, the literature in recent years has been ambivalent about the ability to correctly diagnose fetal growth restriction during the second trimester. Some studies have confirmed the value of second trimester ultrasound in predicting growth discordance, but other studies have not reached this conclusion [13–17]. Therefore, in this study, we aimed to evaluate the accuracy of second trimester ultrasonography to diagnose sFGR, and assess the diagnostic value of indicators related to the diagnosis of sFGR.

## Methods

### Study population

One hundred and eighteen women who had been diagnosed with sFGR in MCDA twin pregnancies at the fetal medicine center from Leiden University Medical Center were selected in the present retrospective study between January 2008 and December 2013. In all participants, monochorionic chorionicity was determined on the 11–14 weeks of gestation ultrasound scan based on a single placental mass with a T‐sign [9]. One hundred and sixty and two MCDA pregnant women who had been diagnosed without sFGR over the same period. We included MCDA pregnant women with two lives fetuses at birth or at onset of labor (≥28 weeks’ gestation) and known fetal outcomes (birth weight). Cases with fetal chromosomes and major structural abnormalities were excluded, as well as those who lacked second trimester ultrasound measurement and birth weight data.

### Ethics statement

After given informed consent, the data of prenatal ultrasound measurements, maternal characteristics, and neonatal outcome of all monochorionic twins born in the center were retrospective recorded in this database. Since the ethical approval and informed consent is not needed for anonymized retrospective studies in the Netherlands, this study was exempt from institutional review board approval.

### Diagnosis of sFGR

In the study, the definition of sFGR after birth was a single birth weight (BW) < 3 rd percentile in a twin, or birth weight discordance (BWD)≥25% between two twins, while the BW of the smaller twin < 10th percentile. BWD was calculated as (Large BW−Small BW) / Large BW×100. Early prenatal diagnosis of sFGR was defined as a single EFW < 3 rd percentile in a twin, or at least 2 of the following 4 parameters must be met (fetal weight of one fetus < 10th percentile, abdominal circumference of one fetus <10th percentile, EFW discordance≥25%, UA pulsatility index (PI) of the smaller fetus > 95th percentile) [18]. The calculation formula of EFW discordance was (Large EFW−Small EFW)/large EFW×100. The cut-off value of EFW discordance was defined as 25%, which represents that the estimated fetal weight discordance between twins exceeds 25%. The calculation formula of AC discordance was (Large AC−Small AC)/large AC×100. The cut-off value for AC discordance is 10%, which means that the discordance in abdominal circumference between twins exceeds 10%.

### Demographic and clinical characteristics

Basic information, including maternal age, parity, gestation, were collected by using the electronic medical records. Ultrasound measurements were performed at 14 to 26 weeks of gestation, which was used to collect the ultrasonographic data [abdominal circumference (AC), estimated fetal weight (EFW), placenta position, deepest vertical pocket (DVP) of amniotic fluid of each fetus, umbilical artery (UA) and ductus venosus (DV) blood flow].

Abnormal UA blood flow was defined as absent/reversed end diastolic flow (AEDF/REDF), or pulsatility index (PI) > 95% must be met [6]. The PI of DV was greater than 95%, which was considered to be abnormal blood flow [19]. We defined the abnormal amount of amniotic fluid as DVP <2cm or DVP> 8cm [20].

### Statistical analysis

We calculated the sample size according to the following formula

$$n={\left(\frac{Z_{1-\alpha /2}}{\delta }\right)}^{2}(1-p)p$$ (1)

$$n={\left[\frac{57.3\times Z_{1-\alpha /2}}{arc\;\sin\left(\delta /\sqrt{p(1-p)}\right)}\right]}^{2}$$ (2)

In the present study, α = 0.05, δ = 0.1. The specificity and sensitivity of ultrasound diagnosis for sFGR were 68.33% and 85.94%, respectively [21]. The number of sFGR group required 95 participants according to the formula (1). The 34 participants in non-sFGR group was required according to the formula (2), The sample size of the two groups both met the research requirement.

Continuous variables were presented as mean ± standard deviation and independent T test was used to compare the differences. Categorical variables are represented by n(%). Pearson’s χ^**2**^ test was used for categorical variables. Then, compared ultrasonographic diagnosis in the second trimester with the diagnosis of sFGR after birth to determine diagnostic effectiveness.

Receiver operating characteristics (ROC) curves were assessed the area under the curve (AUC) as the diagnostic value of ultrasound indicators in MCDA pregnancies with sFGR, the sensitivity and specificity of these independent indicators were computed. All statistical analyses were performed by SPSS version 19.0 software (IBM SPSS Statistics 19). Statistical significance was defined as *P* < 0.05.

## Results

### Maternal characteristics

Two hundred and eighty MCDA twin pregnancies (totally 560 fetuses) were identified and included in the study. Basic maternal characteristics of sFGR group (n = 118) and non-sFGR group (n = 162) were presented in Table 1. The mean age of the participants was 32.06±4.76 years. About 43.9% of pregnant women were primiparas. Of 280 pregnant women, 149 were pregnant at least twice, accounting for 53.2%.

**Table 1:** Maternal characteristics between the two groups.

	Total	sFGR	Non-sFGR	*P-value*
	(n = 280)	(n = 118)	(n = 162)	
**Age** (*years*,*Mean ± SD)*	32.06 ± 4.76	31.89 ± 4.46	32.18 ± 4.97	0.616
**Gestation**				
**<2**	131 (46.8%)	55 (46.6%)	76 (46.9%)	0.960
≥**2**	149 (53.2%)	63 (53.4%)	86 (53.1%)	
**Parity**				
**<3**	188 (67.1%)	81 (68.6%)	107 (66.0%)	0.648
≥**3**	92 (32.9%)	37 (31.4%)	55 (34.0%)	
**Primipara**	123 (43.9%)	49 (41.5%)	74 (45.7%)	0.489
**Anterior placenta**	107 (38.2%)	40 (33.9%)	67 (41.4%)	0.205

Unless indicated otherwise, data are given as n (%).

sFGR, selective fetal growth restriction; non-sFGR, selective fetal growth restriction.

There were no significant differences in maternal age and the proportions of gestation, parity, primipara, and placental position between the two groups (*P* > 0.05).

### Results of the second trimester ultrasound measurements

Table 2 showed the characteristics of the fetal second trimester ultrasound results. Higher prevalence of AC and EFW discordance were observed among118 pairs of twins in sFGR group (83.1% and 75.4%), compared with twins in non-sFGR group (24.1% and 9.9%, *P* < 0.000; Table 2).

**Table 2:** Comparison of ultrasound measurements between two groups of twins.

	Total	sFGR	Non-sFGR	*P-value*
	(n = 280)	(n = 118)	(n = 162)	
AC discordance	137 (48.9%)	98 (83.1%)	39 (24.1%)	<0.001
EFW discordance	105 (37.5%)	89 (75.4%)	16 (9.9%)	<0.001
DVP (Large)				
Normal	196 (70.0%)	83 (70.3%)	113 (69.8%)	0.916
Abnormal	84 (30.0%)	35 (29.7%)	49 (30.2%)	
DVP (Small)				
Normal	151 (53.9%)	56 (47.5%)	95 (58.6%)	0.064
Abnormal	129 (46.1%)	62 (52.5%)	67 (41.4%)	
UA blood flow (Large)				
Normal	271 (96.8%)	116 (98.3%)	155 (95.7%)	0.375
Abnormal	9 (3.2%)	2 (1.7%)	7 (4.3%)	
UA blood flow (Small)				
Normal	175 (62.5%)	48 (40.7%)	127 (78.4%)	<0.001
Abnormal	105 (37.5%)	70 (59.3%)	35 (21.6%)	
DV blood flow (Large)				
Normal	248 (88.6%)	110 (93.2%)	141 (87.04%)	0.094
Abnormal	32 (11.4%)	8 (6.8%)	21 (13.0%)	
DV blood flow (Small)				
Normal	213 (76.1%)	82 (69.5%)	131 (80.9%)	0.028
Abnormal	67 (23.9%)	36 (30.5%)	31 (19.1%)	

Data are given as n (%).

sFGR, selective fetal growth restriction; non-sFGR, selective fetal growth restriction; AC discordance, abdominal circumference discordance; EFW discordance, estimated fetal weight discordance; DVP, deepest vertical pocket of amniotic fluid; UA, umbilical artery; DV, ductus venosus.

With regard to the fetal UA blood flow, the proportion of small fetuses reporting as abnormal was higher in sFGR group than in non-sFGR group (59.3% vs 21.6%, respectively; *P* < 0.001). Among large fetuses, there were 1 case of REDF of umbilical artery and 1 case with absent blood flow in the sFGR group. In the non-sFGR group, 7 cases with AEDF of umbilical artery. In small fetuses, there were 64 twins with AEDF in the sFGR group, 3 cases’ blood flow were reversed, 3 cases of PI> 95%. 30 cases of AEDF in the non-sFGR group, REDF in 2 cases, and PI>95% in 3 cases.

Abnormal DV blood flow were more common in small twins complicated sFGR (*P* < 0.05). There were no significant differences in DVP, large fetal UA, and DV blood flow between the two groups (*P* > 0.05).

### Comparison of the prenatal diagnostic indexes of second trimester ultrasonographic diagnosis and diagnosis of sFGR after birth

By comparing prenatal diagnosis in the second trimester and diagnosis of sFGR after birth as shown in Table 3, 118 pairs of sFGR twins, the second trimester ultrasound accurately identified 89 pairs, and 29 pairs were missed. The result showed the diagnostic effectiveness of second trimester ultrasound is 75.4%. Among pregnant women diagnosed with sFGR by ultrasound, 84% did complicated by sFGR. The missed diagnosis rate and the misdiagnosis rate of the ultrasound measurements were 24.6% and 10.5% respectively. See in Table 4.

**Table 3:** Comparison of prenatal diagnosis and postnatal diagnosis of sFGR.

sFGR	Prenatal diagnosis*		Total
	+	-	
+	89	29	118
-	17	145	162
Total	106	174	280

sFGR, selective fetal growth restriction, diagnosed after birth.

*Prenatal diagnosis was determined by second trimester ultrasonography.

**Table 4:** Diagnostic effectiveness of second trimester ultrasonography for sFGR.

	second trimester ultrasonographic diagnosis
Sensitivity (%)	75.4%
Specificity (%)	89.5%
Positive predictive value (%)	84.0%
Negative predictive value (%)	83.3%
False positive rate (%)	10.5%
False negative rate (%)	24.6%
Positive likelihood ratio	7.2
Negative likelihood ratio	0.3
Youden’s index	64.9%

### Analysis of the accuracy of the prenatal diagnosis of sFGR by using ROC curves

**Fig 1:**
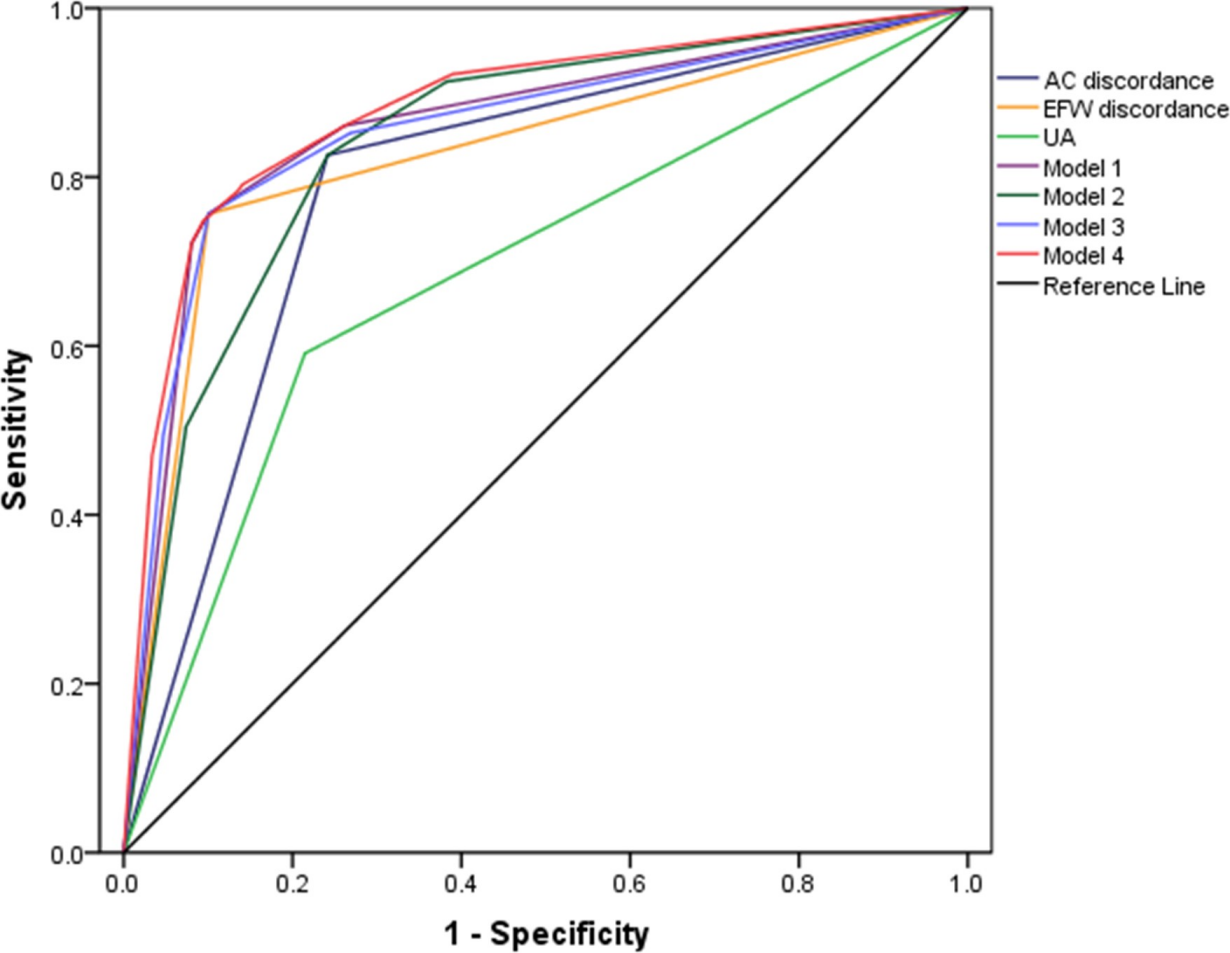
ROC curve analysis of the accuracy of the prenatal diagnosis of sFGR. AC discordance, abdominal circumference discordance; EFW discordance, estimated fetal weight discordance; UA, umbilical artery blood flow in small twins; Model 1, the combination of AC discordance, EFW discordanc; Model 2, the combination of AC discordance, UA; Model 3, the combination of EFW discordance, UA; Model 4, the combination of AC discordance, EFW discordance, and UA.

The accuracy of the prenatal diagnosis of sFGR in twins by the final independent ultrasound indicators were shown in Table 5 and Fig 1. Compared with the other two individual indicators, EFW discordance achieved the highest AUC in diagnosing sFGR (AUC,0.828; 95%CI, 0.774–0.882). When the cut-off value was 10%, the best performance was reached with the AC discordance, in terms of sensitivity. when the cut-off value was set to 25%, EFW discordance reached the highest specificity.

**Table 5:** Analysis of the accuracy of the prenatal diagnosis of sFGR by using ROC curves.

	Sensitivity (%)	Specificity (%)	AUC (95% *CI)*	*P-value*
AC discordance	82.6	75.8	0.792 (0.736–0.849)	<0.001
EFW discordance	75.7	89.9	0.828 (0.774–0.882)	<0.001
UA	59.1	78.5	0.688 (0.622–0.754)	<0.001
Model 1*	75.7	89.9	0.861 (0.812–0.910)	<0.001
Model 2^†^	82.6	75.8	0.843 (0.795–0.892)	<0.001
Model 3^‡^	75.7	89.9	0.858 (0.809–0.907)	<0.001
Model 4 ^§^	74.8	90.6	0.882 (0.839–0.926)	<0.001

AC discordance, abdominal circumference discordance; EFW discordance, estimated fetal weight discordance; DVP, deepest vertical pocket of amniotic fluid; UA, umbilical artery in small twins; DV, Ductus venosus.

* Model 1, the combination of AC discordance, EFW discordance.

^†^ Model 2, the combination of AC discordance, UA.

^‡^ Model 3, the combination of EFW discordance, UA.

^§^ Model 4, the combination of AC discordance, EFW discordance, UA.

The ROC analysis was conducted to reveal the best combination of AC discordance, EFW discordance, and smaller fetal UA blood flow. We derived four combined models of diagnostic indicators, including Model 1 (AC discordance, and EFW discordance) and Model 2 (AC discordance, and small fetal UA), Model 3 (EFW discordance, and small fetal UA), Model 4 (AC discordance, EFW discordance, and small fetal UA). Results proved that these factors were jointly used, the sensitivity or specificity would be improved. Under the optimal cut-off value of model 1 and model 3, they both had the same sensitivity and specificity (75.7%, 89.9%; respectively), but the AUC of Model 1 was better than Model 3 with a smaller advantage (0.861 vs 0.858; respectively). Model 2 had the highest sensitivity but the AUC was poorest. Overall, Model 4 had the highest diagnostic value, with the AUC of 0.882 (95%CI, 0.839–0.926).

## Discussion

In present study, we found that ultrasound measurement in the second trimester is an effective method for early prenatal diagnosis of sFGR. The combination of AC discordance, EFW discordance and the small fetal UA blood flow was the best diagnostic indicator for sFGR.

When providing prenatal care, pregnant women are often divided into low-risk and high-risk groups [20]. Fetal growth restriction is considered a "high risk" in some pregnancy specific diseases [22]. In our study, the ultrasonographic diagnosis between 14 and 26 weeks of gestational age could correctly identify 74.5% of sFGR twins. Of all twins with sFGR detected by ultrasound, 84.0% of fetuses were diagnosed accurately.

The results showed that most of the sFGR twins can be diagnosed by ultrasound during the second trimester, and then these pregnant women with sFGR can be regarded as high-risk pregnancy, prenatal monitoring, appropriate treatment and timely delivery, which could minimize the risk of adverse pregnancy outcomes. From the overall point of view, the ability to accurately diagnose sFGR and non-sFGR was 64.9%. This result was not as high as we expected. Therefore, ultrasonography in the second trimester can only help early prenatal diagnosis of sFGR to a certain extent, and obstetricians still need to be cautious about those who may have mild abnormalities but have not yet been diagnosed.

We assessed three previously described ultrasonographic indicators of sFGR, including AC discordance, EFW discordance and small twin’s UA blood flow. Our analysis suggested that AC discordance would be able to detect 82.6% of MCDA twins at risk for sFGR. A study found that after 14 weeks of gestation, AC was the most important parameter in the prediction of adverse outcomes [13]. In addition, it had been reported by Doulaveris et al. that second trimester AC was associated with an increased risk of FGR [23]. AC has a high sensitivity and specificity, a study has shown that AC is the first biometric index to be affected early in FGR [24, 25]. The reason may be that AC represents the development of internal organs and the nutritional status of the fetuses. When AC decreases, it means fetal poor nutritional status and internal organ dysplasia [26], so we suppose that AC discordance is highly sensitive to the occurrence of sFGR.

Following the documentation by D’Antonio et al. [27], they indicated that EFW discordance was an independent predictor of perinatal outcomes in twin pregnancy. Our study further proved that the discordance of twins’ EFW had diagnostic value of sFGR. Fetal weight is an important index that reflects fetal growth and development in the uterus. The reason for using this parameter as a diagnostic indicator of adverse outcomes was that weight discordance was always present in all twin pregnancies and this kind of circumstance may represent physiological conditions or adaptive changes to the uterine environment [28]. However, on the other hand, this may be the result of a pathological condition involving the fetus or placenta [28]. We speculate that when the twins no longer adapt to the uterine environment or these pathological conditions are further aggravated, not only the EFW discordance between twins increases, but also the risk of sFGR increases.

Our research observed that UA in small twins was an independent ultrasound indicator to diagnose sFGR. The pathological mechanism of sFGR in MCDA pregnancy depended not only on the presence of abnormal placental sharing, but also on the magnitude and direction of blood flow interchange through placental anastomosis [27]. The UA is a major vascular pathway connecting fetus and placenta, supplying nutrition and oxygen for fetal growth and development [29]. If sFGR occurs in the twins, the placenta function will be damaged and blood flow resistance in the UA may increase [30]. A previous study had acknowledged that increased UA vascular resistance was associated with lower fetal growth [31]. Therefore, we believed that the of UA blood flow, especially the increased vascular resistance of UA, could be used as a diagnostic indicator of sFGR.

By comparing ROC curves, it would appear that the diagnostic value of the combination of AC discordance, EFW discordance, and small fetal UA blood flow for MCDA pregnancy complicated with sFGR was the best. This was not an unexpected finding, because this comprehensive combination had suggested that when the advantages of these individual indicators were aggregated, the sensitivity and specificity were both improved, corresponding the diagnostic effectiveness in twins with sFGR was increased.

In this study, we did not exclude the twin-twin transfusion syndrome (TTTS). First of all, at present, there is limited research on the effect of amniotic fluid volume on the measurement of estimated fetal weight in twin pregnancies. Many studies of singleton pregnancy found that amniotic fluid volume did not affect the accuracy of ultrasound measurement of fetal weight [32–35]. There were 69 twins who met the TTTS diagnosis in the present study, of which 26 (22.03%) were in the sFGR group, and 43 (23.63%) in the non-sFGR group. There was no statistical difference between the two groups (χ^2^ = 1.24, *P* = 0.266). In addition, In the sFGR group and the non-sFGR group, there were 18 (69.23%) and 31 (72.09%) twins with TTTS who underwent fetoscopic intervention, respectively, and there was also no significant difference (χ^2^ = 0.11, *P* = 0.765). Further, we implemented sensitive analysis by excluding the 69 twins with TTTS, then, performed the analysis of the diagnostic value of sFGR by the second trimester ultrasound. As we expected, its results showed extremely similar performance to the previous results with twins of TTTS included. The ROC analysis found that the area under the curve of each ultrasound index changed to a very small extent, fluctuating between 0.009–0.059.

Diagnosing sFGR during pregnancy in current clinical surveillance remains challenging. Our study confirmed the importance of second trimester Doppler and ultrasound measurements to diagnosed sFGR in MCDA twins. When the twins complicate abnormal umbilical blood flow or discordant abdominal circumference or discordant estimated fetal weight, we should perform appropriate management according to the severity of the complication and the wishes of the mother and her families. On one hand, we would increase the frequency of ultrasound and Doppler monitoring of pregnant women and expectant management with early preterm delivery. On the other hand, due to the high risk of fetal intrauterine death in sFGR, in order to improve the survival rate of the one twin, surgical treatments such as selective fetal reduction has been an important method in twin pregnancy.

There are several strength of this study. One is that the hospital is a fetal medicine referral center capable of providing fetal treatment and management of complicated MCDA twin pregnancies. It reduces the likelihood that MCDA pregnancy of developing sFGR pursues care at another center, which may be regarded as representative of the general population. Also, Diagnosis and treatment in a single referral center may minimize the variability in clinical examination, which makes the results more credible.

There are some limitations of this study. Firstly, the present study is a retrospective study with limited causal inference. Secondly, missing values for height and weight of the pregnant women restricted us to obtain a complete picture of basic characteristics of pregnancy women. Finally, for the EFW discordance, the confidence intervals of the odds ratio was relatively wide. This variability needs to be investigated in future research with larger sample sizes, allowing the accurate assessment of diagnostic value of EFW discordance.

In conclusion, we found that second trimester ultrasound measurements had an effective value in the early prenatal diagnosis of sFGR. Moreover, we believed that obstetricians and gynecologists should comprehensively consider the three indicators with the greatest diagnostic value for sFGR: AC discordance, EFW discordance, and small fetal UA blood flow, then taking timely interventions to improve fetal prognosis and reduce the incidence of adverse perinatal outcome.

## Supporting information

S1 Data (XLS)
